# Toward a Multi-Omics-Based Single-Cell Environmental Chemistry and Toxicology

**DOI:** 10.1021/acs.est.2c02831

**Published:** 2022-07-12

**Authors:** Jordi Dachs, Maria Vila-Costa

**Affiliations:** Department of Environmental Chemistry, IDAEA-CSIC, c/Jordi Girona 18-26, Barcelona, Catalunya 08034, Spain

**Keywords:** multi-omics, single-cell science, bioconcentration, bioaccumulation, toxicity, pollutant

Increasing resolution has always been a goal for environmental chemistry and toxicology in their quest to expand knowledge on the transport, biogeochemistry, and sinks of natural- and anthropogenic chemicals in the environment, as well as their functions and effects in ecosystem and human health. Linking biology and chemistry is at the core of environmental sciences,^1^ and it is now possible to study comprehensive cell signatures by means of single-cell approaches. The trend toward the analysis of increasingly smaller samples, eventually reaching nanosamples and even single-cell approaches,^1−5^ has resulted in massive data sets. The measures at cellular level, at higher temporal and spatial scale resolution, require significant methodological development, but allows for new questions with eventual knowledge breakthroughs.

The evolution of single-cell science has been favored by its multidisciplinary nature, with many developments occurring at the fringe of its subfields. The emergence of high-throughput multiomic approaches such as genomics, transcriptomics, proteomics, lipidomics, and metobolomics have increased the study of responses at the cellular level. This is innovative and transformative science shaping contemporary environmental toxicology and biogeochemistry of biogenic and man-made chemicals. Their ecosystem counterparts, such as the metagenomics and metatranscriptomics, have allowed a more precise picture of the genes and players involved in the biogeochemistry of nutrients, pollutants, and carbon in the oceans, land, and in anthropic systems, such as wastewater treatment plants. In parallel, high-resolution mass spectrometry has also been developed exponentially during the last decades, allowing the target, suspect, and nontarget analyses of pollutants with unprecedented coverage of the chemical space, accuracy, and precision. Both the multiomic and mass spectrometry techniques have progressed thanks to the concurrent availability and development of big-data approaches, including data mining, chemometrics, bioinformatics, and artificial intelligence approaches, all supported on increasing available computing power. The latter, indeed, allows processing of the millions of sequences of DNA or RNA, or mine the huge data sets of high resolution mass spectra, or sequences, efficiently.

Single-cell multiomic approaches address the intrinsic variability of cell chemistry (DNA, RNA, proteins, lipids, metabolomes, pollutants), instead of that of bulk samples made of multicellular tissues or populations of microorganisms. Such elucidation of the heterogeneity among cells belonging to the same tissues or microbial community is important for disentangling physiological distinct populations or cells groups with specific response signatures to pollutants, or eventually discovering new or rare cells playing a role in pollutant or metabolite transformations. Indeed, these omic signatures are valid indicators of cell-to-cell physiological state, and how it relates to stressors including chemical concentrations, thus contributing to decipher pollutant adverse effects through understanding the adverse outcome pathways.

Analogously, the increasing prevalence of single-cell omic approaches in biomedical sciences has allowed a better understanding of the mechanisms driving pathologies in human diseases, particularly in oncology research.^2,3^ While in biomedical sciences, multiomics single-cell approaches are developing at an exponential pace, environmental science requires additional development of appropriate methodologies for quantification of pollutants at higher cellular resolution. So far, environmental studies on bioaccumulation or transformation of pollutants, or the effects of pollutants on organisms, tissues, or microorganisms has been based on bulk samples, but not on few cells (nanosamples). Such new age of knowledge at the cellular resolution, thanks to single-cell omics, is just starting to emerge in the environmental sciences.^4−10^

Bioconcentration and bioaccumulation play key roles in environmental chemistry, as they relate environmental concentrations in water or sediments to internal organism concentrations, which eventually induce a biological response.^11^ Hydrophobic pollutants accumulate in membranes with concentrations many orders of magnitude higher than in water, but the conceptual overcoming of the “hydrophobic paradigm”, where bioaccumulation is predicted by the octanol–water partition coefficient (*K*_OW_) or other physicochemical descriptors, may only be possible through multiomics at nanoscale, as there are detoxifying mechanisms modifying the ratio of internal/external concentrations, such as degradation and efflux pumps, that are intimately linked to the genes harbored by cells and their activation. Analytical approaches for nanosamples can also be applied to single-particles.^7^ Such approaches would also allow determining bioconcentration factors’ differences between different microorganisms or cells, which can efficiently be sorted with fluorescent, isotopic, or other probes. Eventually, the decrease of knowledge complexity that multiomics at single cell can offer, as a reductive approach to the system, will be invaluable within the adverse outcome pathway, which allows to relate cellular responses to environmental impacts.

Such research goals toward a multiomic environmental chemistry and toxicology with single cell resolution is not free from methodological difficulties and research needs. Obviously, lowering the limits of detection of chemical and omic analyses is mandatory. The single-cell environmental chemistry requires a major development in the manipulation and processing of nanosamples. So far, these analytical methods have generally been thought for bulk samples, but a single cell approach requires the analyses of a large number of samples, in order to assess cellular heterogeneity, which can only be obtained with a miniaturization of sample handling and an automation of laboratory procedures able at working with small volumes.^12^ Such miniaturization approaches will be also a step toward application of green chemistry principles.^13^ Data treatment pipelines for deciphering the coupling of pollutant concentrations with multiomic information will also need a substantial effort, and the development and application of novel data science procedures may be needed. This knowledge will also result in better models of cellular responses as a more mechanistic view is attained. Such advancements, from laboratory procedures to data fusion, will allow building of bridges among disciplines and subfields, facilitating the linkage between biology and chemistry, with the envisaged synergies needed for disruptive advances and new conceptual frameworks.
